# Evaluating the cyclic ratio schedule as an assay of feeding behaviour in the European starling (*Sturnus vulgaris*)

**DOI:** 10.1371/journal.pone.0206363

**Published:** 2018-10-23

**Authors:** Jonathon Dunn, Clare Andrews, Daniel Nettle, Melissa Bateson

**Affiliations:** Centre for Behaviour and Evolution & Institute of Neuroscience, Newcastle University, Newcastle, United Kingdom; CNRS, University of Strasbourg, FRANCE

## Abstract

The cyclic ratio (CR) schedule is a behavioural assay developed to study feeding in rats, in which the number of operant responses required to obtain food reward (the ratio requirement) increases and then decreases in a repeating cycle. In a recent study, we used the CR schedule with European starlings (*Sturnus vulgaris*) to investigate the effects of an early-life manipulation on adult feeding behaviour. As this was the first time the CR schedule had been used with any bird species, a more in-depth evaluation is warranted. Here, we performed a fuller CR experiment with the same birds as the prior study, a year later. First, we examine the individual consistency of feeding behaviour between experimental sessions and also between CR schedules comprising different ratio requirement progressions. We found that between-session consistency was poor to moderate, and that a geometric ratio progression provided greater between-session consistency than an arithmetic ratio progression. Second, we tried to replicate some of the canonical findings from rats working on CR schedules. In contrast to findings from rats, we found that defence of feeding rates did not increase when starlings were acutely food deprived. However, as in rats, we found that the post-reinforcement pause increased linearly with the upcoming ratio requirement, suggesting that starlings were able to learn the cyclic nature of the schedule. Third, we compared the results from the present study concerning the impacts of our early-life treatment with those from our earlier study. We found that the majority of our previous findings were replicated in the same individuals one year on, reinforcing our previous conclusion that the early-life manipulation had canalised our birds into two groups with different patterns of feeding rate defence.

## Introduction

The cyclic ratio (henceforth ‘CR’) schedule is a behavioural assay that was developed to study the operant regulation of feeding in rats [1,2]. Briefly, the procedure involves presenting experimental subjects with a series of cyclically ascending and descending work requirements (so-called ‘ratio requirements’) to obtain a food reward. Two independent behavioural variables can be extracted from the responses of animals working on a CR schedule (henceforth collectively ‘CR feeding behaviour’). The first is the feeding rate independent of ratio requirement (henceforth ‘preferred feeding rate’), and the second is the extent to which the rate of food intake changes as the ratio requirement increases (henceforth ‘defence of feeding rate’). Using a theoretical model, Staddon [1] predicted that defence of feeding rate would be increased by acutely depriving animals of food, or maintaining them below free-feeding body weights. He also predicted that preferred feeding rate would be increased by making food more palatable. Both predictions were corroborated by subsequent experimental work in rats [2,3]. Since its development nearly 40 years ago, the CR schedule has been largely used by researchers as a behavioural assay to understand how various pharmacological manipulations affect feeding in rats [4,5].

In a recent study conducted in 2016, we used a CR schedule with a cohort of adult European starlings (*Sturnus vulgaris*) to obtain measures of individual differences in preferred feeding rate and defence of feeding rate [6]. These measures were then related to an early-life hand-rearing manipulation [7] in which we independently varied the amount of food received (henceforth Amount; either Plenty or Lean), and the duration of begging effort per day (henceforth Effort; either Hard or Easy) over a ten day period in a 2 x 2 cross-factored design. In Dunn et al. [6], each experimental subject was exposed to a daily CR schedule session for two consecutive days. Within each session we presented the following sequence of ratio requirements: 2, 4, 6, 8, 10, 12, 12, 10, 8, 6, 4, 2; and this cycle was repeated up to five times per session for a maximum of three hours. We found effects of our developmental treatments on both defence of feeding rate and preferred feeding rate. To the best of our knowledge, this was the first time a CR schedule has been used with starlings, or indeed any bird. For this reason and those outlined below, we felt that a more in-depth exploration of the CR schedule as a behavioural assay of feeding in starlings was warranted.

Our first reason relates to methodology. One of the purported advantages of the CR schedule is that in rats exposed to a single experimental CR session, CR feeding behaviour for each component ratio requirement is comparable to that provided by more time-intensive, fixed ratio schedules in which only a single ratio is programmed in each session [2]. This implies that there is consistency between different daily CR sessions (although session-to-session consistency is seldom, if ever reported in CR rat studies, with little justification usually given for the number of sessions used). However, we did not test for session-to-session consistency in our original study. We also used a sequence of ratio requirements that increased in an arithmetic progression (i.e. ratio requirement successively increasing by 2, rather than doubling); however, many other studies have used geometric progressions [8,9] and the possible ramifications of our choice are unclear. Furthermore, to the best of our knowledge, the only studies that have explicitly compared the behaviour of animals working on arithmetic vs. geometric ratio progressions have been for progressive ratio and not CR schedules [10].

Second, in our original experiment, we did not determine whether we were able to replicate in the starling canonical findings of rodents working on CR schedules. These canonical findings are as follows: food deprivation affects defence of feeding rate, but not preferred feeding rate; and the post-reinforcement pause (time between the delivery of food reinforcer and first peck of the next trial, hereafter termed ‘PRP’) increases linearly with upcoming ratio requirement. Ettinger and Staddon [2] presented this second finding as a key demonstration that rats are able to detect and anticipate the variation in ratio requirement during the CR schedule.

Our final reason relates to the long-term stability of individual differences in feeding behaviour. In Dunn et al. [6], we found that early-life treatments affected adult CR feeding behaviour. Specifically, larger amounts of food in early-life (Plenty treatment) increased preferred feeding rate, whilst higher amounts of begging (Hard treatment) produced stronger defence of feeding rate. The effects of our developmental manipulation were particularly striking given that it lasted for only ten days in the first fortnight post-hatching and the Dunn et al. [6] experiment occurred two years later when the birds were adult. A limitation of our original CR experiment was that it represented a brief snapshot of adult feeding behaviour, and so an important question is whether the differences in feeding behaviour we found in 2016 [6] were still present in 2017 when the same birds were one year older.

To investigate these issues and evaluate the CR schedule as an assay of feeding behaviour in starlings, we administered a series of CR tasks to the same cohort of birds used in Dunn et al. [6] one year later with the objective to: 1) establish the extent to which individual differences in CR feeding behaviour are consistent from session to session; 2) examine the effect of ratio progression type (arithmetic versus geometric) on individual consistency in CR feeding behaviour; 3) determine whether acute food deprivation causes starlings to increase their defence of feeding rate, but not their preferred feeding rate; 4) determine whether PRP increases linearly as a function of upcoming ratio requirement; and 5) determine whether the results of Dunn et al. [6] concerning the impacts of early-life treatments were replicated when the birds were one year older.

## Methods

### Ethical statement

The study adhered to ASAB/ABS guidelines for the use of animals in research. Birds were taken from the wild under Natural England permit 20121066 and the research was completed under Home Office licence PPL 70/8089, with approval of the Animal Welfare and Ethics Review Board at Newcastle University. After the completion of the current experiment the birds were retained in the laboratory for further studies.

### Subjects and housing

Our subjects were from a cohort of 32 wild-caught, hand-reared European starlings (16 male, 16 female, determined genetically) that were removed from nest boxes five days after hatching in 2014. These birds contained eight families of four siblings matched for weight on day five. Next, they experienced a ten day developmental manipulation, in which two factors, Amount and Effort, were varied independently [7]. All birds were given nine feeds per day. The Amount treatment manipulated the total food received: those in the Plenty groups were fed to satiation on each feed, whereas those in the Lean groups were given a mean of 73% of the amount given to the corresponding Plenty group on their most recent feed. The Effort treatment manipulated amount of begging: the nests of the Easy groups were visited just for the nine feeds per day, whereas those in the Hard groups had an additional nine ‘sham’ visits, on which they were stimulated to beg for the approximate duration of a feed (2 mins) without food being delivered. Thus, nestlings in the Hard groups begged for 36 minutes per day, compared to 18 minutes per day in the Easy groups. Our two developmental treatments were delivered in a 2 x 2 factorial design, resulting in the following four groups: Plenty Easy (PE), Plenty Hard (PH), Lean Easy (LE) and Lean Hard (LH). As the results of the genetic sexing were not available until the development treatment had begun, sex ratios were unavoidably uneven between groups (male:female ratios: PE = 4:4, PH = 1:7, LE = 8:0, LH = 3:5).

Following the end of the manipulation at 15 days post-hatching, birds were fed to satiation on every feed until fledging around day 21. Subsequently, birds were kept in mixed-treatment cages with ad libitum food until they had all been observed feeding themselves where they were released into two indoor aviaries (215 x 430 x 220cm; ~18C, 40% humidity; 15L:9D light cycle) in mixed-sex groups of no more than 20 birds on day 56 with *ad libitum* food as before. They were maintained in non-breeding condition by the constant light cycle of long days.

For the current experiment birds were moved to individual cages (100 x 45 x 45cm with two perches and plastic baths, and the same light cycle, temperature and humidity conditions as the aviaries) when they were approximately three years of age. Two birds died before they were 20 months old (one male LE bird died in an accident and one female LH bird died due to unknown causes) and so 30 birds were available for both the present experiment and the previous operant experiment in Dunn et al. [6].

### Operant experiment

The current experiment commenced when the birds were 978–1044 days old. Birds were moved to individual operant cages in sets of eight (except where bird missing due to death), keeping natal families together thereby balancing testing order across developmental treatments. Cages were fitted with panels with three illuminable pecking keys and a feeder trough connected to a pellet dispenser delivering 45mg grain-based rodent pellets [11]. Operant panels were controlled remotely using the Whisker Experimental Control system [12]. Birds were habituated to cages and trained to peck keys for pellets prior to the current experiment following the protocol outlined in Feenders and Bateson [11].

Our experiment had three phases (Table 1), each with a slightly different operant task comprising a sequence of discrete-trials ratio schedules. In all tasks, a trial started with illumination (in amber) of the central pecking key. The bird was required to peck the lit key a number of times specified by the current ratio requirement. Successful completion of this requirement within 1800 s resulted in the key light extinguishing, release of 1 food pellet and illumination of the feeding trough for 1 s. Failure to complete the requirement in time resulted in the key light extinguishing and the trial ending. All trials ended within an inter-trial interval of 1 s. Across all phases, operant sessions began at 07:00 (1 h after lights coming on) and ended at 10:00 where *ad libitum* food (domestic chick crumb) and baths were provided. Baths were removed at 17:00 in preparation for the following morning’s session, as was food depending on deprivation treatment (see below).

**Table 1:** Summary of operant experimental timeline.

Phase	Schedule and progression	Day	Deprivation treatment
1	CRA	1	Deprived
		2	Deprived
2	CRG	1	Deprived
		2	Satiated
		3	Satiated
		4	Deprived
3	VR	1	Deprived
		2	Deprived

CRA, cyclic ratio schedule with arithmetic ratio progression; CRG, cyclic ratio schedule with geometric ratio progression; VR, variable ratio schedule. Across each day the maximum number of trials completed was 60.

In phase 1, birds were given two days of a CR task with a sequence of ratio requirements that increased and decreased according to an arithmetic progression (hereafter ‘CRA’; sequence: 2, 4, 6, 8, 10, 12, 12, 10, 8, 6, 4, 2) identical to that in Dunn et al. [6], allowing us to address session-to-session consistency and replication of Dunn et al. [6] (objectives 1 and 5). As in Dunn et al. [6], birds were food-deprived for 14 h (from 17:00 to 07:00) prior to the experimental session. In phase 2, birds received four days of a CR task with a sequence of ratio requirements that increased and decreased according to a geometric progression (hereafter ‘CRG’; sequence: 2, 4, 8, 16, 32, 32, 16, 8, 4, 2), allowing us to address the impact of the type of progression (objective 2). Here, birds were satiated for the middle two days but food deprived for the rest (Table 1), ensuring deprivation treatment was counterbalanced by order. For the middle two days of phase 2, the birds were food-deprived for 14 hours prior to the session as in phase 1 (henceforth, ‘deprived’ days), but for the first and fourth days, food was withdrawn just 1 hour prior to the session (henceforth ‘satiated’ days). Comparison of the two deprived and two satiated days of phase 2 thus allowed us to address the effect of food deprivation (objective 3). To verify that our deprivation treatment had the intended effect, we measured whether the amount of ad lib food eaten between the end of the previous session and the removal of the ad lib food was greater on satiated than deprived days. It was (linear mixed model: β_satiated_ = 3.80, SE = 0.30, LRT = 110.60, p<0.001). Thus, we can be confident that the birds had eaten more prior to the session on satiated than deprived days.

In phase 3, birds were exposed to two days of a variable ratio (VR) task that was identical in every aspect to the phase 2 CRG task that occurred on deprived days, with the exception that ratio requirements were presented in a random, instead of cyclical order. The purpose of this VR task was to act as a control to the CRG task, allowing us to address whether birds learned the cyclic nature of the schedules (objective 4).

Across all phases each daily session was terminated after 60 trials had been completed or after 3 h had elapsed (whichever came first). Thus, birds were exposed to a maximum of 120 trials per bird in phase 1, 240 trials per bird in phase 2 and 120 trials per bird in phase 3. The schedule parameters were chosen to ensure the birds did not become satiated [13,14]. For all operant tasks, the interval between the illumination of the key and the final peck to complete the programmed ratio requirement was collected for every trial and is hereafter termed ‘trial latency’. We also collected the interval between the illumination of the key and first key peck, which was the PRP.

### Data analysis

#### Examining CR feeding behaviour

As in Dunn et al. [6], we measured defence of feeding rate as the slope of the relationship between ratio requirement and trial latency (flatter slope indicates stronger defence); and preferred feeding rate as the average latency to complete trials pooled across all ratio requirements. Staddon [1] suggests two alternative measures to extract from CR data (defence of feeding rate as the slope between feeding rate and peck rate; and preferred feeding rate as the feeding rate where no pecks are required). However, in Dunn et al. [6], we established that the simpler measures we used are highly correlated with those suggested by Staddon. This is also true in the present data (analysis not shown). Hence we report only the simpler measures in this paper.

#### Statistical modelling

All data analyses were conducted in R version 3.3.2 [15]. The raw data files and the R script are available at the Zenodo repository [16]. Note that all latencies (trial and PRP) were log-transformed prior to analysis.

Consistency of CR feeding behaviour (objectives 1 and 2) was compared by calculating the intraclass correlation coefficients (ICC) and their 95% confidence intervals using the ‘irr’ package [17] based on a two-way, single-score intraclass correlation consistency model. For all ICC analyses, the outcome variables were the defence of feeding rate and preferred feeding rate, and the units of analysis were individual birds. We interpreted ICC values as follows (after [18]): 0.75–1 was excellent, 0.6–0.74 was good, 0.4–0.59 was fair and <0.4 was poor.

For testing our predictions about the effect of deprivation (objective 3), whether the birds learned the cyclic nature of the schedules (objective 4) and replicating our findings concerning developmental treatments (objective 5), we constructed Linear Mixed Models (LMMs) using the lmer package [19]. Details of these models are given in the Results section below. All models presented here gave satisfactory distribution of residuals, hence a Gaussian error structure was assumed throughout.

We also extracted parameter estimates and standard errors from our replication analyses, which were entered into fixed-effects meta-analyses using the rma function from the ‘metafor’ package [20]. This was to investigate the possibility that there might be small effects, not significant in any one of the experiments considered individually, but detectable when the information from our current 2017 study were combined with those of the 2016 study reported in Dunn et al. [6]. More generally, it was to establish what the accumulated weight of evidence from all the experiments was regarding the question of developmental treatments and feeding behaviour (objective 5).

## Results

### Objective 1: Daily consistency of CR feeding behaviour

Between-session preferred feeding rates were of good consistency (ICC = 0.68, 95% CI = 0.42 to 0.84), but between-session defence of feeding rates were of poor consistency (ICC = 0.12, 95% CI = -0.26 to 0.46) in the CRA task (days 1 and 2 of phase 1). We found a similar picture when a CRG progression was used (days 1 and 4 of phase 2), with between-session preferred feeding rates of good consistency (ICC = 0.73, 95% CI = 0.51 to 0.86), but between-session defence of feeding rates of fair consistency (ICC = 0.44, 95% CI = 0.10 to 0.69). Defence of feeding rates derived from CRG progressions showed more between-session consistency than those derived from CRA progressions.

### Objective 2: Consistency of CR feeding behaviour across ratio requirement progression type

Next, we combined the data from the two successive days of CRA (days 1 and 2 of phase 1), and combined the data from the two successive deprived days of CRG (days 1 and 4 of phase 2), and analysed to what extent the CRA and CRG phases produced feeding parameters that were consistent with each other. Preferred feeding rates showed excellent consistency (ICC = 0.78, 95% CI = 0.59 to 0.89), between CRA and CRG progressions. Contrastingly, defence of feeding rates showed poor consistency (ICC = 0.18, 95% CI = -0.18 to 0.51) between CRA and CRG progressions (days 1 and 2 of phase 1 versus days 1 and 4 of phase 2).

### Objective 3: Effect of deprivation on defence of feeding rates

We sought to replicate Ettinger and Staddon’s [2] finding in rats that food deprivation affected defence of feeding rate and hence, we constructed an LMM where the outcome variable was trial latency and the fixed effects were deprivation status (deprived or satiated), ratio requirement, and the interaction between deprivation status and ratio requirement. Also included was a random effect of bird ID within natal nest. This LMM was used to compare two days of the CRG task (thereby using the same ratio requirements as the Ettinger and Staddon [2] study with rats) where the birds were satiated (days 2 and 3 of phase 2) to two days of the same task where the birds were food deprived (days 1 and 4 of phase 2), pooling the data from each deprivation treatment across days. If the canonical prediction that deprivation affects defence of feeding rate but not preferred feeding rate is supported, then there should be a significant interaction between deprivation and ratio requirement, but no main effect of deprivation on ratio requirement.

**Fig 1:**
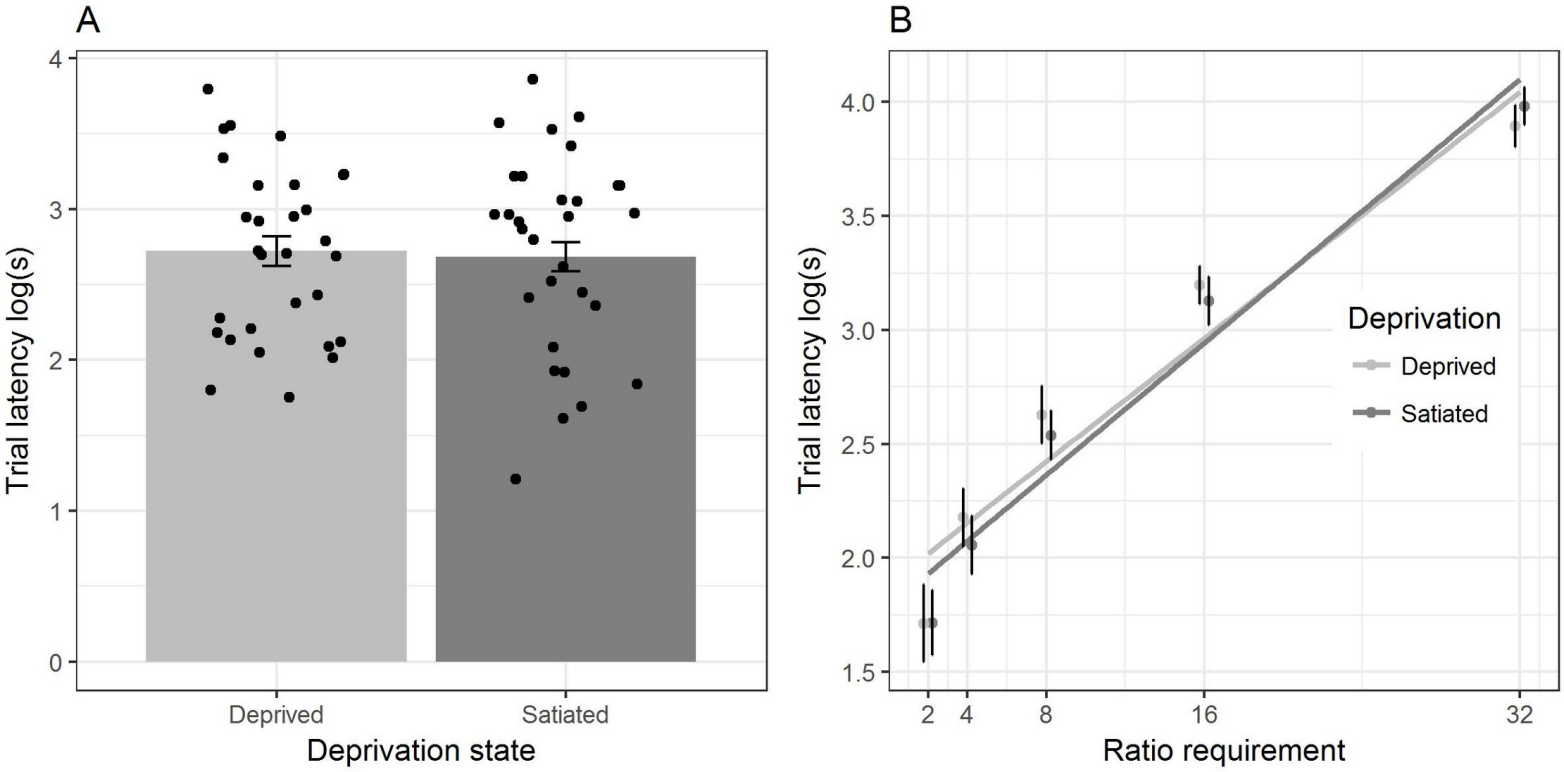
No effect of food deprivation status on CR feeding behaviour. A) Bars show mean individual trial latency, hence preferred feeding rate, plus between-bird SE, by the two levels of the deprivation treatment. Note: all ratio requirements are pooled and individual bird latencies are overlaid as points. B) Individual mean and between-bird SEs of trial latency by ratio requirement (pecks required to complete a trial) by the two levels of the deprivation treatment. The slope of the regression line indicates the strength of defence of feeding rate (flatter line, stronger defence). All panels are based on raw data from 30 birds; note CRG data are shown only to ensure direct comparability with the Ettinger and Staddon study [2] with rats.

We found no significant interaction between food deprivation treatment and ratio requirement (β = 0.01, SE = 0.003, LRT = 1.81, p = 0.18; Fig 1B), indicating that food deprivation did not differentially affect defence of feeding rate when ratio requirements were increased. Additionally, food deprivation did not affect trial latency, and hence preferred feeding rate (β_satiated_ = -0.04, SE = 0.04, LRT = 1.01, p = 0.32; Fig 1A). As expected, ratio requirement was positively related to trial latency (β = 0.07, SE = 0.002, LRT = 1224.96, p<0.001).

### Objective 4: Effect of ratio requirement on PRP

We sought to replicate the linear relationship between PRP and upcoming ratio requirement used by Ettinger and Staddon [2] as evidence that rats learned and were able to anticipate upcoming ratio requirements in a CR schedule. To do this we used the time to initiate each trial, which was the PRP for the trial that had directly preceded it.

Consequently, we constructed three LMMs where the outcome was PRP, with the first containing upcoming ratio requirement as a fixed effect, the second containing previous ratio requirement as a fixed effect and the third containing the intercept only (null model). In all models we included bird ID nested within natal nest as a random effect. We did this using two days of deprived CRG data (days 1 and 4 of phase 2, thereby using the same ratio requirements as the Ettinger and Staddon [2] study with rats) and compared our findings when two days of deprived VR data (days 1 and 2 of phase 3) were used. By randomising the ratio requirements that appeared in cyclical order in phase 2, the VR schedule acted as a control as birds had no way of detecting and anticipating the upcoming ratio requirement.

**Fig 2:**
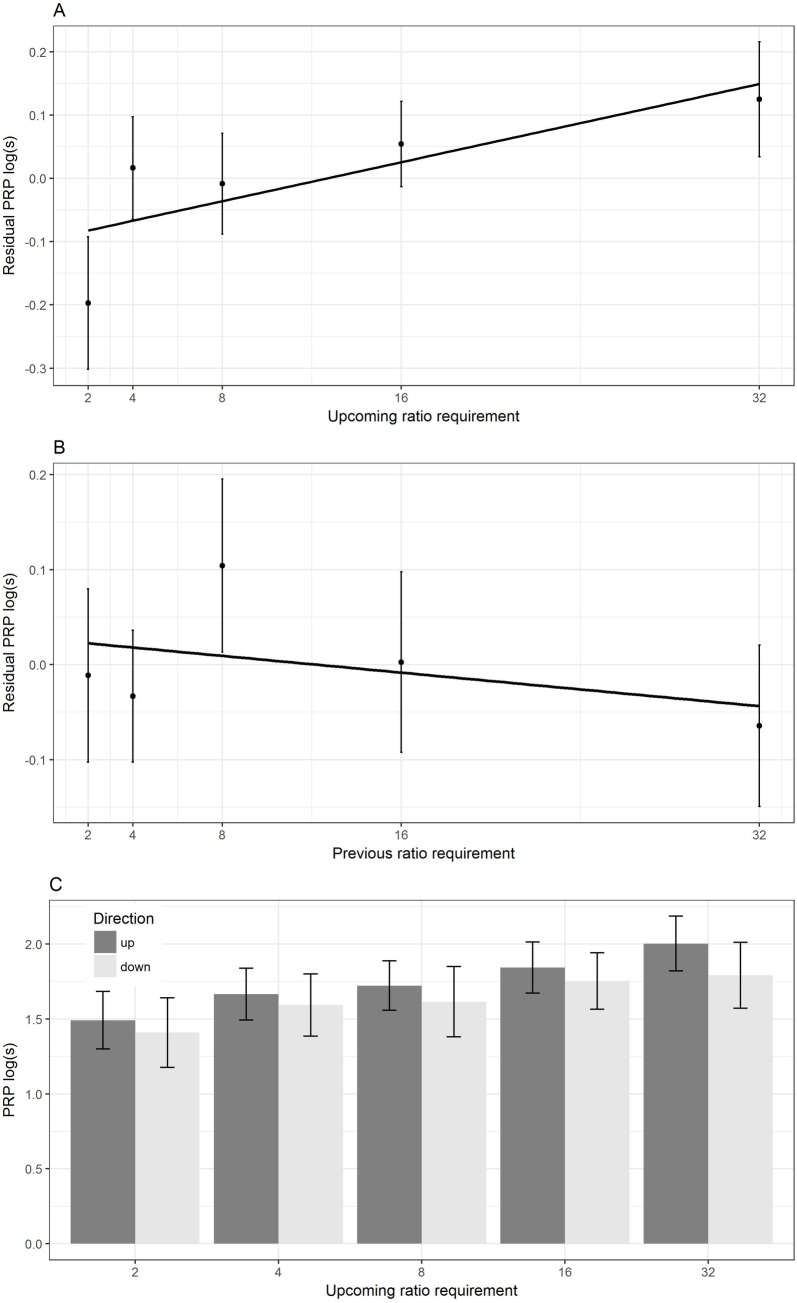
Positive relationship between upcoming but not previous ratio requirement and PRP. A) Individual mean and between-bird SEs of PRP by upcoming ratio requirement (pecks required to complete a trial). B) As A) but by previous ratio requirement. Note: for clarity we present PRP as residual PRP for panels A) and B). This is because both previous and upcoming ratio requirement may act together to influence PRP, making interpretation of the raw data difficult. Thus, residual PRP was calculated for panel A) by extracting residuals from a regression of logged PRP against previous ratio requirement. Residual PRP was calculated for panel B) by extracting residuals from a regression of logged PRP against upcoming ratio requirement. C) Individual mean PRP plus between-bird SEs by upcoming ratio requirement (pecks required to complete a trial). Data were split by whether the ratio requirement came in ascending (up direction, represented by black filled bars) or descending (down direction, represented by grey filled bars) part of the cyclical sequence. The plots represent raw data from 30 birds; note CRG data are shown only to ensure direct comparability with the Ettinger and Staddon study [2] with rats.

We found that when ratio requirements were presented cyclically (i.e. the CRG schedule), the model containing upcoming ratio requirement better explained PRP compared to the model containing previous ratio requirement or the null model, as measured by Akaike’s corrected information criterion (ΔAICc: upcoming = 0.0; previous = 8.6; and null = 9.9). There was a statistically significant, positive association between upcoming ratio requirement and PRP (LMM with upcoming ratio requirement as predictor: β = 0.01, SE = 0.004, LRT = 11.95, p<0.001; Fig 2A), but not between previous ratio requirement and PRP (LMM with previous ratio requirement as predictor: β = 0.007, SE = 0.004, LRT = 3.36, p = 0.07; Fig 2B). In contrast, when the sequence of ratio requirements was presented in a random order (i.e. the VR schedule), models containing either upcoming ratio, previous ratio or the null model were equally poor at explaining PRP (ΔAICc: upcoming = 0; previous = 0.8 and null = 1.7). Here, there were no statistically significant associations between either upcoming ratio requirement and PRP (LMM with upcoming ratio requirement as predictor: β = -0.006, SE = 0.004, LRT = 2.78, p = 0.10), or previous ratio requirement and PRP (LMM with previous ratio requirement as predictor: β = 0.004, SE = 0.004, LRT = 1.13, p = 0.29). Thus, taken together our findings support the idea that birds anticipated the upcoming ratio requirement in the CRG but not the VR schedule.

Another way of addressing the same question is to ask whether the trial being the ascending rather than the descending part of the cyclic sequence affected the linear relationship between PRP and upcoming ratio requirement. This is because the previous ratio differs according to which part of the cycle the trial is in, but the upcoming ratio does not. To investigate this, we repeated our model that used upcoming ratio requirement as a predictor of PRP for the cyclic data, but also included an interaction between ratio requirement and position (ascending/descending). We found that whether a given ratio requirement was in the ascending or descending part of the cycle did not influence PRP (interaction between ratio requirement and position: β = 0.004, SE = 0.07, LRT = 0.37, p = 0.54; Fig 2C). Thus, this again supports the prediction that birds anticipated the upcoming ratio requirement in the CRG but not the VR schedule.

### Objective 5: Replication of the impacts of early-life treatments on feeding behaviour

The findings from Dunn et al. [6] we sought to replicate concerned the effects of the developmental treatments on CR feeding behaviour. To do this, we identified the predictor variables that had a high relative importance from the model selection and averaging carried out in Dunn et al. [6]. These variables were then used to construct three LMMs with trial latency as the outcome variable. All models contained the following predictors: food Amount, begging Effort, ratio requirement and the interaction between begging Effort and ratio requirement. We also included a random effect of bird ID nested in natal nest. The only difference between models was the dataset used: the first used the original CRA data from Dunn et al. [6] (as that experiment was carried out in 2016, we henceforth refer to this as the 2016 CRA data), the second used the CRA data (days 1 and 2 of phase 1) from the current experiment (2017 CRA) and the third (2017 CRG) used the CRG data from the current experiment where the birds were deprived (days 1 and 4 of phase 2), respectively pooling data across days.

**Fig 3:**
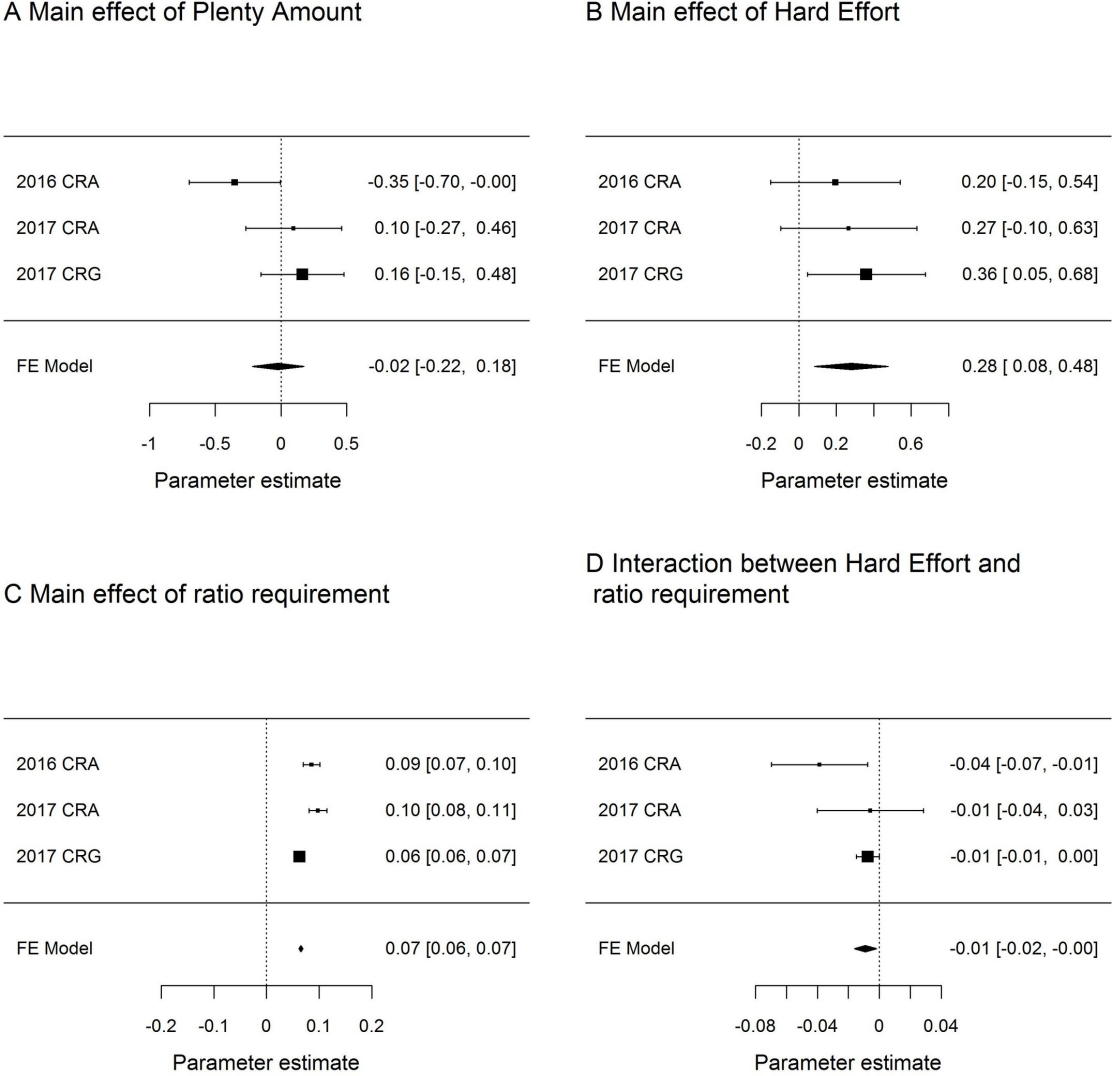
Forest plots from meta-analyses across 2016 CRA, 2017 CRA and 2017 CRG datasets. Sub-panels show the effects of the following on logged trial latency: a) Plenty Amount; b) Hard Effort; c) ratio requirement; d) interaction between ratio requirement and Hard Effort. Shown are the central estimates of effect size and their 95% confidence intervals. FE Model stands for ‘Fixed Effects’ model.

We replicated some, but not all of our previous findings from Dunn et al. [6], depending on the exact data that was used (Fig 3). First, we replicated the pattern in the 2016 CRA data that defence of feeding rate was stronger for Hard birds than Easy birds, but this was only when the 2017 CRG data was used (β(Hard) = -0.01, SE = 0.004, LRT = 3.78, p = 0.05; Fig 3D). In contrast to the 2016 CRA findings, we did not find any effect of food Amount on the preferred feeding rate in either 2017 CRA or 2017 CRG (Fig 3A). Also, when the 2017 CRG data was used, Hard birds had lower preferred feeding rates (β(Hard) = 0.36, SE = 0.16, LRT = 4.55, p = 0.03; Fig 3B), which we did not find when the CRA data was used, either from 2016 or 2017.

When the replications were meta-analysed, the 95% confidence intervals for most parameter estimates did not overlap with zero. Significant parameter estimates were begging Effort (Fig 3B), ratio requirement (Fig 3C) and the interaction between ratio requirement and begging Effort (Fig 3D). Only food Amount was not significant (Fig 3A). The direction for all meta-analysed summary parameters was the same as in the analysis reported by Dunn et al. [6]. Thus, in summary, it appears that our previous finding that the Amount treatment affected preferred feeding rate may have been a false positive (i.e. type I error), but there does appear to be a robust effect of the Effort treatment on both preferred feeding rate, and the defence of feeding rate.

## Discussion

We administered a series of operant tasks to provide an evaluation of the CR schedule as an assay for feeding behaviour in European starlings. Specifically our experiment sought to: 1) establish the extent to which individual differences in CR feeding behaviour are consistent from session to session; 2) examine the effect of ratio progression (arithmetic (CRA) versus geometric (CRG)) on individual consistency in CR feeding behaviour; 3) determine whether food deprivation caused starlings to increase their defence of feeding rate but not preferred feeding rate; 4) determine if PRP increased linearly as a function of upcoming ratio requirement; and 5) replicate the results of Dunn et al. [6] concerning the impacts of early-life treatments on feeding behaviour, when the same individual birds were one year older. How our results relate to each of these objectives is discussed in further detail below.

We studied two kinds of consistency in CR feeding behaviour. Session-to-session consistency describes the extent to which individuals perform in a similar way in successive daily sessions of exactly the same schedule (objective 1). Schedule consistency describes the extent to which behaviour of individuals under one schedule (CRA) is consistent with their behaviour under the other (CRG) (objective 2).

We found that individual differences in CR feeding behaviour had poor to good between-session consistency. This suggests that contrary to what is implied by Ettinger and Staddon’s findings with rats [2], a single CR session is not enough to provide consistent measures of CR feeding behaviour in starlings. The fact that later studies with rats [8,21] have used anywhere between 14 to 45 daily sessions may reflect the need to average out session-to-session variation in performance, and so it is probable starlings may also require a similar period of time. Thus, it would appear that the early promise of the CR schedule as a quick behavioural assay has been overstated. Future work should focus on whether stable CR behaviour can be achieved in starlings and if so, how many sessions of training on the ratio are required for stable behaviour to emerge. We also found that between-session consistency was greater for preferred feeding rates than defence of feeding rates. Interestingly, between-session consistency was greater for defence of feeding rates derived from a CRG progression than a CRA progression. One possible explanation for this finding is that in a CRG progression, the range of ratio requirements measured is broader, which may act to stabilise our estimate of defence of feeding rate.

We also found that across different ratio schedule progressions, defence of feeding rate consistency was poor, but preferred feeding rate consistency was excellent. This was surprising for two reasons: first, other studies with rats suggest that barring ratio strain (a disruption of operant responding that occurs as a result of large ratio size and/or low reinforcement frequency), CR feeding behaviour ought to be relatively insensitive to the ratio requirement values comprising the CR schedule [21]. Second, as our CRG progression included larger ratio requirements than our CRA progression, we expected that between-progression consistency of preferred feeding rates would be worse than for defence of feeding rates. One difficulty in interpreting our results is that in our experiment, the CRG progression is confounded with larger maximum ratio requirements. We therefore cannot tell whether any differences between CRA and CRG are due to the progression itself, or the maximum ratio requirement value contained. This issue notwithstanding, our results seem to suggest that CRG progressions provide more consistent measures of CR feeding behaviour in starlings at least, and that measures of CR feeding behaviour are sensitive to the ratio requirement progression used.

Contrary to Staddon’s [1] predictions and subsequent, canonical experimental work in rats [2], we did not find that defence of feeding rate was affected by food deprivation status. Why our deprivation treatment did not have its intended effect is not clear, as we found that our starlings consumed significantly more ad libitum food prior to the operant session in the satiated treatment relative to the deprived treatment. Given that our birds were housed in an open economy and had access to food outside experimental sessions, we might have expected the principle of ‘inelastic demand’ to apply to our deprived birds e.g. [22]; thus, when non-experimental food was made scarce, deprived birds ought to have minimised the loss of overall consumption by increasing their defence of feeding rate during our CR experiments. One possibility is that our satiation treatment was not sufficiently long and that our birds were still behaving as if they were food deprived. Another possibility is that the poor daily consistency of the defence of feeding rates derived from the CRG progression acted to obscure our results. Although defence of feeding rate was not affected by deprivation status, neither were preferred feeding rates, suggesting that some of our results were in line with Staddon’s predictions and results. Further work needs to focus on better manipulating satiation in starlings before we can determine whether the CR schedule can be used to distinguish between the effects of different behavioural variables on feeding behaviour in the same manner as with rats [4,5,8,21,23].

Our analysis of PRP yielded a more positive picture. Here, we found that PRP increased linearly with ratio requirement, but this reflected upcoming and not previous ratio requirement. Moreover, this effect disappeared when the component ratio values were presented in a random, not cyclical order. We also found that position in the cyclic sequence (ascending/descending) did not influence PRP. Taken together, our results support Ettinger and Staddon’s [2] assertion that subjects working on a CR schedule learn to anticipate the upcoming ratio requirements. This is somewhat surprising as our starlings were only exposed to two experimental CR sessions and we found that between-session CR feeding behaviour consistency was generally poor. Further work examining within-ratio and within-session behaviour may help elucidate whether our result truly does reflect learning and an ability to anticipate upcoming ratio requirement, or whether there is an alternative, yet unconsidered explanation.

In addition to replicating some of the canonical results found with rodents working on the CR schedule, we tried to replicate the results found in Dunn et al. [6] to see whether the differences in feeding behaviour by developmental history that we originally found had persisted or attenuated in the same birds one year later. Encouragingly, we were able to replicate the majority of our results when meta-analysed. Specifically, we were able to replicate our original finding that birds which experienced the Hard begging Effort developmental treatment had stronger defence of feeding rate as adults. However, we were not able to replicate our original finding that larger Amounts of early-life food increased adult preferred feeding rates. Instead, we found it was birds that experienced the Hard begging Effort developmental treatment were the ones that had decreased preferred feeding rates, which is something that was not significant in the original experiment considered alone. Overall, all meta-analysed parameter estimates were consistently in the same direction as in Dunn et al. [6]. Thus, our results suggest that many of the key differences we found in Dunn et al. [6] have persisted one year on, further supporting our previous suggestion that our early life manipulation had canalised our birds into two groups with different patterns of feeding rate defence.

In conclusion, we feel that the CR schedule can be used as a behavioural assay to study some aspects of feeding in starlings, but further exploration needs to be undertaken to discover the limits of its usefulness in comparison to other techniques, such as deriving the breakpoint for animals working on a progressive ratio schedule [10,24]. First and foremost, future work needs to focus on whether greater consistency in CR feeding behaviour can be achieved between experimental sessions and if so, how long this takes for behaviour to stabilise. Where possible, CRG progressions should be used in order to maximise the between-session consistency of defence of feeding rate. Care must be taken to ensure that sufficient component ratio requirements are available to measure defence of feeding rate and that the largest ratio requirement in the chosen CRG progression does not cause ratio strain. Future work should also focus on better manipulating food deprivation and/or altering the palatability of the food reinforcers to test whether some of Staddon’s other canonical results hold true for starlings working on a CR schedule. While it appears that starlings do anticipate the upcoming ratio requirements in the CR schedule, it is unclear whether this truly reflects learning and the length of time needed to learn the schedule. Nevertheless, the CR schedule still appears to provide a useful means of capturing variation in feeding rate defence between individual starlings.
